# International Normalized Ratio as a Screening Test for Assessment of Anticoagulant Activity for Patients Treated With Rivaroxaban or Apixaban: A Pilot Study

**DOI:** 10.3389/fphar.2019.01177

**Published:** 2019-10-08

**Authors:** Fanny Ofek, Dana Barchel, Nofar Perets, Tomer Ziv-Baran, Ahmad Mahajna, Talia Filipovich-Rimon, Osnat Garach-Jehoshua, Maya Berlin, Matitiahu Berkovitch

**Affiliations:** ^1^Pharmacy Department, Assaf Harofeh Medical Center, Affiliated to Sackler School of Medicine, Tel-Aviv University, Tel-Aviv, Israel; ^2^Internal Department, Assaf Harofeh Medical Center, Affiliated to Sackler School of Medicine, Tel-Aviv University, Tel-Aviv, Israel; ^3^Institute for Drug Research, School of Pharmacy, The Hebrew University of Jerusalem, Jerusalem, Israel; ^4^Department of Epidemiology and Preventive Medicine, School of Public Health, Sackler School of Medicine, Tel-Aviv University, Tel-Aviv, Israel; ^5^Division of Hematology, Assaf Harofeh Medical Center, Affiliated to Sackler School of Medicine, Tel-Aviv University, Tel-Aviv, Israel; ^6^Clinical Pharmacology and Toxicology Unit, Assaf Harofeh Medical Center, Affiliated to Sackler School of Medicine, Tel-Aviv University, Tel-Aviv, Israel

**Keywords:** rivaroxaban, apixaban, INR, anti-FXa activity, direct oral anti- FXa anticoagulants, anti-FXa chromogenic assay

## Abstract

**Introduction:** In patients treated with direct oral anti activated factor X (anti-FXa) anticoagulants such as apixaban and rivaroxaban, there are several emergency and non-emergency conditions in which anticoagulation activity should be measured. The validity of the common global clotting tests, prothrombin time and international normalized ratio (PT/INR) for determination of blood levels of these drugs, has been widely investigated. As the anticoagulation activity evaluation “calibrated anti-FXa” of these drugs is relatively more expensive and less available, we aimed to build a prediction model for anticoagulation activity assessment based on INR values.

**Methods and Findings:** One hundred sixty samples from 80 hospitalized patients treated with apixaban or rivaroxaban were tested using PT/INR and Anti-FXa chromogenic assay. Two blood samples, trough and peak, were collected from each subject. Participants were randomly divided into two equal groups. One group (n = 40) was used to build the model, which was validated by the second group (n = 40). There was a strong correlation between anti-FXa concentrations and INR in rivaroxaban treated patients (r = 0.899, p < 0.001). Therefore, we were able to build a formula for rivaroxaban patient group which reliably represent the relationship between these two parameters. The correlation in apixaban treated patients was less predictive (r = 0.798, p < 0.001) and the formula suggested could not be validated.

**Conclusions:** In our study, we developed a formula that estimates the anticoagulant activity of rivaroxaban by obtaining INR values. Where anti-FXa assay is unavailable, our proposed formula may be considered as a screening test for rivaroxaban.

## Introduction

The direct oral anticoagulants (DOACs), apixaban and rivaroxaban, inhibit activated factor X (FXa) in a reversible manner. Apixaban (twice daily) and rivaroxaban (once daily) are indicated for prevention of stroke and systemic embolism in patients with non-valvular atrial fibrillation (NVAF). They are also indicated for the prevention of venous thromboembolism (VTE) in patients undergoing elective hip or knee replacement surgery, as well as for treatment of acute deep vein thrombosis (DVT) or pulmonary embolism (PE), and prevention of recurrent DVT or PE in patients at continued risk for recurrent DVT and/or PE. Rivaroxaban, 2.5mg twice daily, co-administered with acetylsalicylic acid (ASA) alone or with ASA plus clopidogrel, is approved in Europe for the prevention of atherothrombotic events in adult patients after an acute coronary syndrome (ACS) with elevated cardiac biomarkers (Kreutz et al., 2017). The same rivaroxaban vascular dose (2.5mg twice daily) combined with low dose aspirin has been recently registered for the prevention of atherothrombotic events in adult patients with stable coronary artery disease or symptomatic peripheral artery disease at high risk of ischaemic events. This regimen may be considered in carefully selected patients who are at high risk of cardiovascular events and low risk of bleeding who do not require therapeutic anticoagulation or dual antiplatelet therapy for another indication (Eikelboom et al., 2017).

Apixaban and rivaroxaban present a more favorable pharmacokinetic profile than warfarin and have a very wide on-therapy window. Thus, monitoring coagulation routinely is not usually required to guide dosing. However, the use of a reliable assay to assess anti-FXa activity may be needed in several clinical conditions such as major bleeding or thrombotic events, need for an urgent procedure, AF patients presenting with acute ischemic stroke prior to administration of thrombolytic therapy, potential drug–drug interactions, impaired renal or hepatic function, and suspected overdosing or incompliance (Ofek et al., 2017). Assessment of the anticoagulation activity in special populations to avoid intracranial hemorrhage or major bleeding due to overdose may also be desirable. Special populations include elderly, low body weight, obese, and post bariatric surgery patients. Administration of these drugs as crushed tablets *via* a nasogastric tube may also raise questions regarding the extent of absorption (Ikeda and Tachibana, 2016). Furthermore, as specific antidotes are presently available, anti-FXa measurement might be helpful to evaluate the necessity of their administration to prevent overuse of these expensive medications (Bauer, 2015).

Intuitively, prothrombin time (PT) and international normalized ratio (INR) were suggested as methods for measuring the anticoagulation effect of anti-FXa drugs. Indeed, clot-based assays such as PT/INR are rapid, widely available, and inexpensive. However, coagulation factor abnormalities and levels of plasma proteins or other drugs may affect these tests and contribute to deranged results. In addition, these tests show diverse responses to different FXa inhibitors. This variation may be due to chemical or physical interactions between a specific FXa inhibitor and phospholipids present within the thromboplastin reagents (Barrett et al., 2010).

There is also a significant variability in laboratory results due to diversity of the reagent/instrument used. The Belgian national External Quality Assessment Scheme (EQAS) performed two nationwide surveys using lyophilised plasma samples spiked with rivaroxaban (Van Blerk et al., 2015) and apixaban (Van Blerk et al., 2017) to assess the effect of these drugs on routine coagulation assays: PT, activated partial thromboplastin time (aPTT), fibrinogen, and antithrombin. The laboratories used many different reagent/instrument combinations. Rivaroxaban prolongs the PT in a concentration-dependent manner. However, PT reagents vary markedly in their sensitivity to rivaroxaban and the PT has insufficient sensitivity to exclude on-therapy levels of rivaroxaban (Tripodi et al., 2015). For example, among PT reagents, Neoplastin R^®^ was the most sensitive to rivaroxaban while Innovin^®^ and Thromborel S^®^ were the least sensitive (Van Blerk et al., 2015). The difference in sensitivity may derive from the reagents composition.

Using different reagents showed only minor influence on PT results when apixaban was tested since PT is barely affected by apixaban. The survey results (Van Blerk et al., 2017) confirm and extend previous studies that PT reagents are much less sensitive to apixaban than to rivaroxaban (Patel et al., 2015; Ofek et al., 2017). This difference probably derives from a slower binding of apixaban to FXa (Tripodi et al., 2015). Most PT reagents are insufficiently sensitive even to detect apixaban’s above on-therapy levels (Skeppholm et al., 2015; Bonar et al., 2016; Samuelson and Cuker, 2017). aPTT is less sensitive than the PT to factor FXa inhibitors and in general, does not play a role in the laboratory assessment of anticoagulation with these agents (Francart et al., 2014; Dale et al., 2014).

The data presented by these surveys (Van Blerk et al., 2015; Van Blerk et al., 2017) provide useful information for clinical laboratories and assist clinicians to properly interpret coagulation tests.

The INR is affected by interlaboratory variability. This variability may derive from a difference between the international sensitivity index (ISI) as specified by the manufacturer and the ISI determined in the independent calibration. According to the guidelines issued by the working group of the International Society on Thrombosis and Haemostasis, sets of calibrant plasmas should be checked and validated before being applied to local calibration. This validation requires that the INR obtained for fresh patient plasma, calculated using the calibration carried out by means of the set of calibrant plasmas, should not differ by more than 10% from the INR calculated using the calibration recommended by the World Health Organization (WHO) (Van den Besselaar et al., 1999). Nevertheless, a proficiency testing surveys for DOACs performed by the Italian Federation of Thrombosis Centers as part of the EQAS activity resulted in a conclusion that measurement of the DOAC plasma concentration in a real world situation is reliable and that the interlaboratory variability is low and similar to that of the INR (observed within the same survey). These results show that INR variability is small and can be as reliable as the DOAC plasma concentration measurement (Tripodi, 2018).

Variability, lack of precision between laboratories using different thromboplastin reagents, and low sensitivity, pose a question regarding the utility of these clinical tests for measuring the pharmacodynamics effects of the anti-FXa inhibitors (Barrett et al., 2010).

In contrast to the marked differences between these two agents regarding the PT/INR test, calibrated assays of anti-FXa exhibit a high degree of linear correlation (r^2^ = 0.83–1.0) within the on-therapy range for both apixaban and rivaroxaban (Samuelson and Cuker, 2017). Anti-FXa chromogenic assay calibrated with drug-specific standards has been proved to have excellent linearity with the plasma concentration of direct FXa inhibitors (Samama et al., 2010a; Samama et al., 2010b; Harenberg et al., 2011; Douxfils et al., 2012; Douxfils et al., 2013). The anti-FXa assay is a better technique with greater sensitivity and less variability than the clot based global tests for assessing patient’s coagulation status. It has been used for evaluation of the anticoagulation intensity of and for drug quantification in patients treated with direct FXa inhibitors (Barrett et al., 2010). Recently, the DaXa-inhibition assay was proposed (Van Pelt et al., 2018). The authors advocate a drug-effect based approach in the anti-FXa medication management, which enables to replace all drug specific assays by one general assay, a universal anti FXa assay that measures the direct inhibitory effect of all anti-FXa drugs. Implementation of such an assay would possibly allow a more widespread use mainly by reducing costs and laboratory management difficulties.

Although calibrated anti-FXa activity assay is the common practice test for measurement of rivaroxaban and apixaban, it is currently relatively more costly and still not broadly available in medical settings. We saw it extremely tempting to investigate integration of the common, prompt, and inexpensive global tests (i.e. PT/INR) into clinical practice as an alternative to anti-FXa assays in urgent medical conditions or when a definitive assessment is not obligatory. For this purpose, we designed a study aimed to examine the existence of a possible correlation between the PT/INR and chromogenic anti-FXa activity assays measurements in hospitalized patients treated with rivaroxaban or apixaban.

## Methods

Assaf Harofeh Medical Center is a 900-bed university affiliated hospital in central Israel, serving a population of approximately one million people. The hospital provides major services including emergency, intensive care, general medical, surgical, cardiac, pediatric, neonatal, gynecologic, and obstetric services. This prospective observational study was approved by the local institutional review board and was conducted on hospitalized patients treated with rivaroxaban or apixaban.

## Study Population and Study Design

Patients aged 18 years and above admitted to the internal medicine wards in our institution from October 2017 through April 2018 and treated with rivaroxaban or apixaban, either newly started or on continuous therapy for NVAF or for prevention and treatment of DVT/PE, were recruited. A minimum of 4 days of dosing was selected to ensure that both agents reached steady state (Frost et al., 2013; Frost et al., 2014). The exclusion criteria were liver disease (defined as cirrhotic patients with Child Pugh B and C classification), an estimated creatinine clearance of less than 30 ml/min, concomitant use of drugs documented to interact with rivaroxaban/apixaban, clinically significant active bleeding, high dose therapy of rivaroxaban/apixaban during the acute phase of DVT/PE, patients with active malignancies and patients not capable of signing the informed consent. Lupus anticoagulants can influence PT and lead to INRs that do not accurately reflect the true level of anticoagulation. In our study, patients with antiphospholipid antibody syndrome (APS) were not excluded. However, none of our patients had APS. Eligibility of patients was determined according to the inclusion or exclusion criteria and a written informed consent was obtained from all patients before enrollment. Two blood samples, trough and peak, were collected from each participant. Rivaroxaban and apixaban are rapidly absorbed with maximum concentrations (Cmax) appearing 2 - 4 h after tablet intake (Bayer Pharma, 2018; Bristol-Myers Squibb, 2018). Thus, the trough sample was drawn in the morning right before the administration of the anticoagulant and the peak sample was obtained 3 h after the documented drug administration time. Patients receiving rivaroxaban with anticipated study doses (15, 20 mg) had to take the drug right after breakfast as the drug’s absorption for these tablet doses is almost complete after food ingestion. This was done in order to maximize absorption of the drug and to achieve uniform results across the study. As apixaban’s bioavailability is not affected by food (Bristol-Myers Squibb, 2018; Frost et al., 2013), participants were allowed to take the drug with no relation to meal. PT/INR and anti-FXa calibrated for rivaroxaban or apixaban were determined.

Participants were randomly divided into two equal groups. One group was used to build the model (derivation cohort) and the second group was used to validate it (validation cohort). The following data were collected mainly from the patient’s electronic medical records: demographic data (age, gender), weight, body mass index (BMI), blood tests included white blood count, hemoglobin, platelets, liver enzymes, bilirubin, albumin, serum creatinine and glomerular filtration rate calculated with the Modification of Diet in Renal Disease formula, indication for anticoagulant use (AF, DVT and PE), and other medications used on admission and comorbidities. The blood samples were collected in 3.2% sodium citrate tubes. The PT/aPTT were determined by using PT-Fibrinogen HSPLUS and Hemosil Syntahsil reagents (Instrumentation Laboratory) respectively on a coagulation analyzer ACL TOP-500 (Instrumentation Laboratory). The direct FXa inhibitor concentrations (e.g. rivaroxaban, apixaban) were measured in human citrated plasma on ACL TOP-500 analyzer by using chromogenic assay Hemosil Liquid anti-FXa kit (Instrumentation Laboratory) after utilizing rivaroxaban or apixaban calibration curves prepared by specific rivaroxaban and apixaban calibrators (Hyphen Biomed). The anti –Fxa activity measurement was expressed as plasma concentrations in ng/mL. The plasma concentration of rivaroxaban can be also estimated by using the formula: 1 IU/ml = 225 ng/ml (Ikeda and Tachibana, 2016).

## Statistical Analysis

Categorical variables were reported as number and percentage. Continuous variables were evaluated from normal distribution using histogram and Q-Q plot and reported as median and interquartile range (IQR) or as mean and Standard Deviation (SD).

Background characteristics of the derivation and validation cohorts were compared using independent sample t-test or Mann-Whitney test for the continuous variables and chi-square test or Fisher’s exact test were used for the categorical variables.

The peak and trough values were combined in order to analyze the full range of anti FXa and to build one simple-to-use equation for the whole range.

Association between INR and Anti FXa was observed using Spearman’s correlation coefficient. The prediction model was built on the derivation cohort and then, was evaluated using the validation cohort. Curve estimation was performed taking into account linear, logarithmic, inverse, quadratic, power, compound, s-curve, cubic, power, compound, s-curve, growth, and exponential models.

The model was chosen based on the highest R^2^ and was evaluated to meet the assumption of the linear regression (linear association, normal distribution of the residuals, and homoscedasticity).

Root Mean Square Error (RMSE) was used to measure the differences between the observed and predicted anti-FXa values. Bland and Altman plot was used to describe the differences between the observed and estimated values of anti-FXa. A two-tailed P >0.05 was considered statistically significant. The analyses were performed with SPSS version 25.0 (IBM SPSS Statistics for Windows, IBM Corp., Armonk, New York, USA, 2017).

## Results

### Clinical Characteristics of the Participants

Altogether, 96 patients were enrolled for the study. Sixteen were excluded from further analysis due to the following technical reasons: six patients did not consume rivaroxaban after breakfast since they had to fast before a certain procedure, five patients were released from the hospital before we managed to withdraw a second sample, three patients undergone procedures during the time the second sample was about to be taken, one patient was diagnosed with cancer during his current hospitalization and one blood sample volume was insufficient to perform the anti-FXa test. The remaining 80 patients included 40 in each drug treatment group, which was further divided randomly into two groups, derivation and validation cohorts. **Table 1** presents the patients characteristics of rivaroxaban and apixaban treatment groups on admission and comparison between derivation and validation cohorts.

**Table 1:** Rivaroxaban and apixaban treatment groups. Patient characteristics on admission and comparison between derivation and validation cohorts.

	Rivaroxaban group				Apixaban group			
Characteristic^a^	Study Population (n = 40)	Derivation (n = 20)	Validation (n = 20)	P Value	Study Population (n = 40)	Derivation(n = 20)	Validation (n = 20)	P Value
**Age (y)**	77.2 (10.3)	75.8 (11.2)	78.7 (9.5)	0.398	77.5 (70.5–81.7)	77.5 (70.5–79.7)	77.5 (70.5–85)	0.64
**Male sex**	20 (50%)	10 (50%)	10 (50%)	>0.999	9 (22.5%)	6 (30%)	3 (15%)	0.451
**Weight (kg)**	86 (67.7 – 97.0)	90 (65.5 –100.0)	83.5 (68.0 –95.0)	0.573	80 (73–92)	80 (72–110)	80.5 (75.5 – 91.5)	0.879
**BMI (kg/m2)**	29.4 (26.6 – 35.6)	29.3 (25.7 – 38.4)	29.6 (26.7 – 33.2)	0.599	31.2 (25.4–34.4)	30.4 (25.4–33.4)	32.2 (25.7 – 35.4)	0.531
**Scr (mg/dl)**	0.95 (0.76 –1.2)	0.98 (0.72 – 1.2)	0.93 (0.8 – 1.3)	0.968	1.0 (0.8–1.3)	1.0 (0.7–1.1)	1.0 (0.8 – 1.3)	0.231
**GFR (ml/min)**	68 (50 – 82.3)	60 (42.5 – 80.7)	69.0 (52.2 – 83.7)	0.478	58 (43–75.7)	61 (40.5–78.2)	57 (46.7 – 74.7)	0.738
**INR** c **(trough)**	1.6 (1.4 – 1.8)	1.6 (1.3 – 1.8)	1.6 (1.4 – 1.8)	0.799	1.3 (1.2–1.5)	1.4 (1.2–1.6)	1.3 (1.2 – 1.4)	0.529
**INR** c **(peak)**	3.29 (2.76 – 3.76)	3.29 (2.7 – 3.8)	3.29 (2.8 – 3.8)	0.529	1.4 (1.3–1.6)	1.5 (1.3 – 1.7)	1.4 (1.3 – 1.6)	0.213
**PT** c **(s) (trough)**	21.1 (18.5 – 23.1)	21.2 (17.7 – 24.0)	20.7 (18.8 – 23.0)	0.779	18.1 (16.9–19.9)	18.6 (17.0–20.7)	17.9 (16.8–19.3)	0.512
**PT** c **(s) (peak)**	39.4 (33.8 – 44.4)	39.4 (33.3 – 44.9)	39.7 (34.8 – 44.4)	0.547	19.2 (17.4–21.7)	19.6 (17.5–22.4)	18.6 (17.3 – 21.2)	0.195
**aPTT** c **(s) (trough)**	33.4 (30.7-36.2)	32.1 (29.7 – 35.3)	34.7 (31.0 – 36.5)	0.114	31.6 (29.7–34.2)	32.9 (19.7–36.2)	31.3 (29.7 – 33)	0.127
**aPTT** c **(s) (peak)**	40.1 (36.9 – 43.9)	38.6 (35.5 – 41.9)	40.7 (38.0 – 45.0)	0.221	32.5 (30.8–35.6)	33.2 (31.3–37.2)	31.6 (30.4 – 33.6)	0.016
**Anti-Xa** b^,^^c^ **(ng/ml) (trough)**	61.4 (26.9 – 87.3)	58.0 (27.1 – 120.2)	62.5 (26.9 – 78.1)	0.659	122.5 (85.2 – 207.6)	95.1 (72.4–154)	169.2(106.9 – 215.6)	0.06
**Anti-Xa** b^,^^c^ **(ng/ml) (peak)**	327.4 (278.6 – 417.3)	326.7 (278.6 –444.2)	329.9(274.9 – 390.3)	0.989	178.9 (106.9–260.5)	152.5 (90.3–234)	194.8 (136.4 - 278)	0.056
**WBC (K/ul)**	8.8 (6.4–10.6)	8.7 (6.2 – 10.1)	8.8 (6.5 – 11.0)	0.841	7.6 (6.6–9.3)	7.8 (6.6–9.3)	7.2 (6.4–9.3)	0.779
**Hb (g/dl)**	11.6 (10.9-13.4)	11.6 (10.5–13.1)	11.7 (10.9 – 13.5)	0.678	11.3 (10.4–12.6)	11.2 (10.9–13.2)	11.3 (10.1–12.5)	0.383
**Platelets (K/ul)**	185.5 (145.2 – 257.0)	183 (144.5 – 239.5)	185.5 (147.7 – 274.0)	0.414	226 (164.7—272.7)	224 (176–271.5)	227 (145–287.7)	0.718
**Bilirubin (mg/dL)**	0.59 (0.41–0.78)	0.36 (0.58 – 0.71)	0.65 (0.43 – 0.86)	0.270	0.45 (0.28-0.78)	0.46 (0.24–0.87)	0.39 (0.28-0.73)	0.363
**Albumin (g/L)**	36 (5.1)	35.4 (5.9)	36.6 (4.3)	0.458	37.5 (31–40.7)	39 (33–41)	35 (29–41)	0.285
**ALT (U/L)**	16.5 (12.7 – 27.7)	17 (13.7 –27.7)	16.0 (12.0 – 32.2)	0.675	16.5 (11.2–28.0)	17 (11–25)	15 (11.5-30)	0.778
**AST (U/L)**	17.0 (14.0 – 25.0)	15.0 (14.0 – 23.0)	19.0 (15.2 – 28.7)	0.158	18.5 (14.2–25.7)	21 (14–26)	16 (14.5–24)	0.433
Indication for anticoagulant - no. (%)								
**AF**	40 (100%)	20 (100%)	20 (100%)	NA	37 (92.5%)	19 (95%)	18 (90%)	0.487
**DVT**	0 (0%)	0 (0%)	0 (0%)		1 (2.5%)	1 (5%)	0 (0%)	
**PE**	0 (0%)	0 (0%)	0 (0%)		2 (5%)	0 (0%)	2 (10%)	

Abbreviations: BMI, Body Mass Index; Scr, Serum creatinine; GFR, Glomerular Filtration Rate; INR, International Normalized Ratio, PT, Prothrombin Time; aPTT, activated Partial Thromboplastin Time; WBC, White Blood Cells; Hb, Hemoglobin; ALT, Alanine aminotransferase; AST, Aspartate aminotransferase; AF, Atrial Fibrilation; DVT, Deep Vein Thrombosis; PE, Pulmonary Embolism.

a Continuous variables are expressed as mean (SD) or median (IQR). Categorical variables are reported as number and percentage.

b Drug’s anticoagulant activity is expressed as drug concentration ng/ml.

c In order to present one simple model, all sample range (peak and through) was included in a single prediction model.

There was no significant difference between patients in the derivation and validation cohorts in any of the background parameters (**Table 1**).

In 13 and 19 blood samples, the concentration was lower than 30 and 50 ng/mg respectively. In These Samples the Median INR Were 1.28 (IQR, 1.26-1.46; 95%CI, 1.1-1.77) and 1.28 (IQR 1.25-1.48; 95% CI, 1.1-1.77), Respectively.

### Rivaroxaban Group

**Figure 1:**
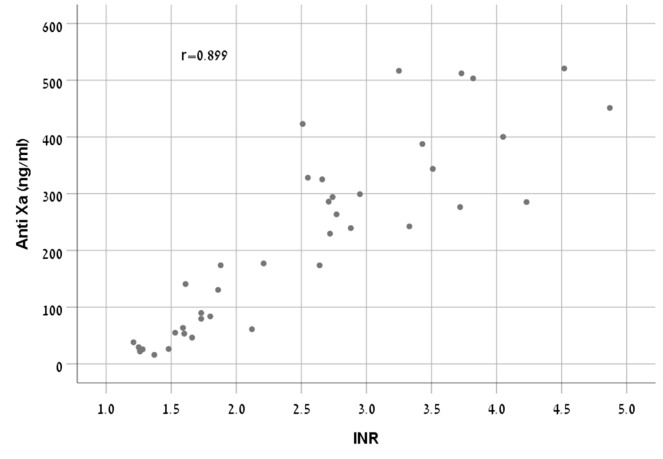
Correlation between INR and Anti-FXa in the rivaroxaban treated group.

There was a strong correlation between anti-FXa concentrations and INR (r = 0.899, p < 0.001) (**Figure 1**).

**Figure 2:**
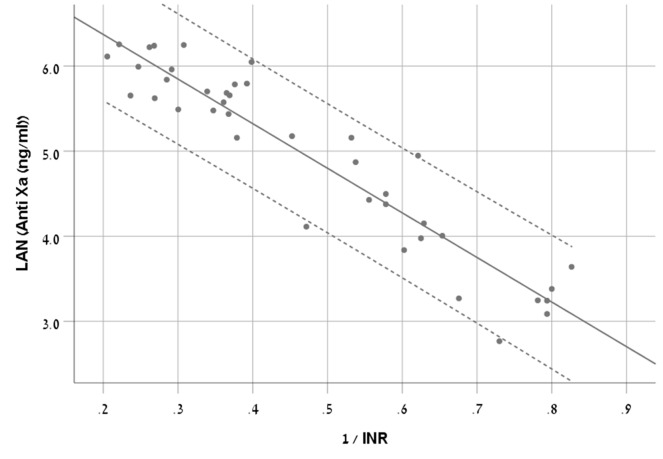
A scatter plot demonstrating the new equation with 95% CI in patients treated with rivaroxaban.

We chose S-curve model as it had the highest R^2^ (0.881) (Equation 1, **Figure 2**).

$$\text{Anti-FXa}(\text{ng}/\text{ml})=e^{7.419-5.241x\frac{1}{\mathrm{INR}}}$$

The RMSE of the model was 77ng/ml in the derivation cohort and 67ng/ml in the validation cohort.

**Figure 3:**
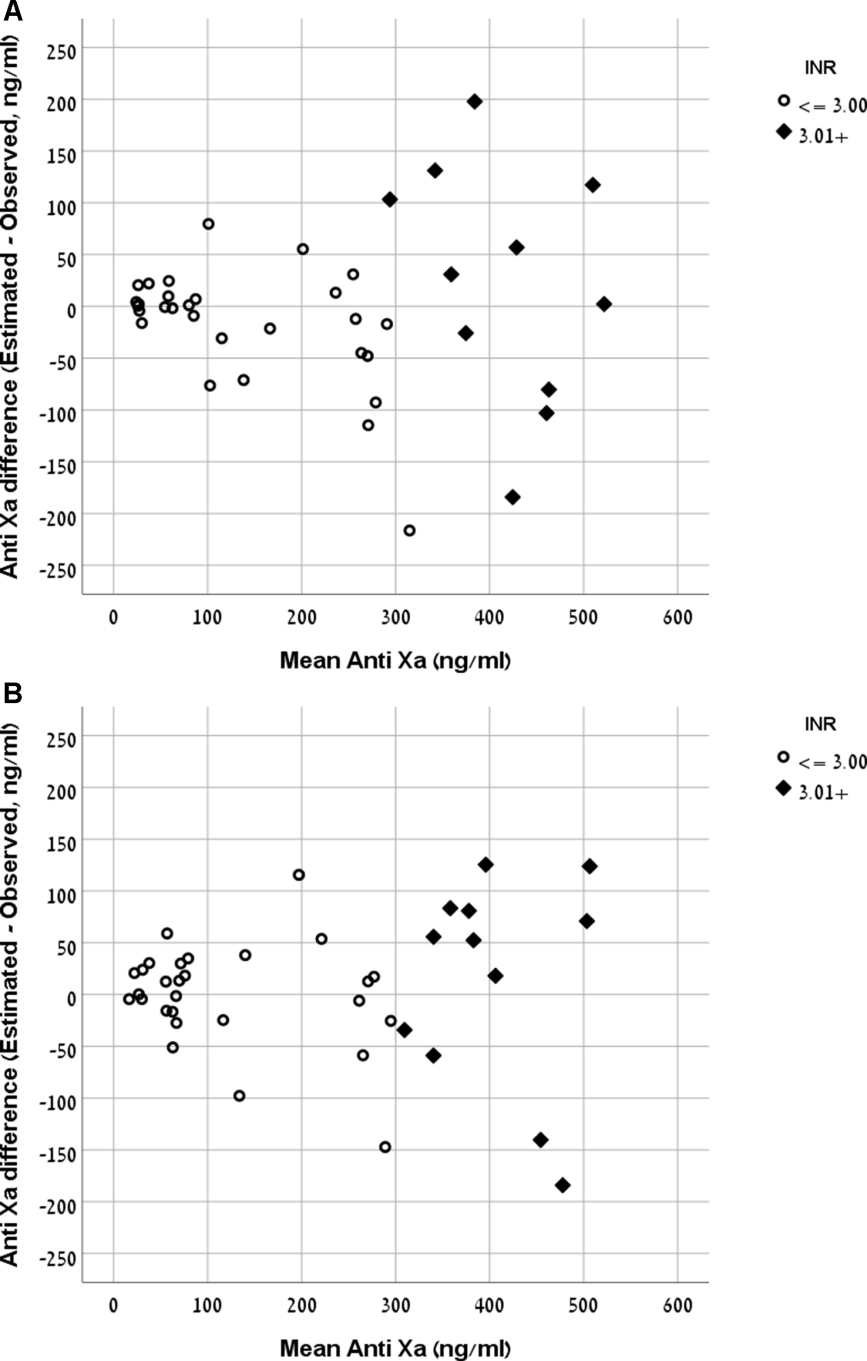
**(A)** Bland and Altman plot demonstrating the mean value of observed and expected versus the difference between them in the rivaroxaban derivation cohort. **(B)** Bland and Altman plot demonstrating the mean value of observed and expected versus the difference between them in the rivaroxaban validation cohort.

The Bland and Altman plot of the derivation and validation cohorts are presented in **Figures 3A, B**, respectively. As clearly shown in these plots, the highest residuals of the models were observed in patients with INR values higher than three. Thus, from a practical point of view, in real life, we marked the blood samples with INR >3 in both the derivation and the validation curves. Our equation refers to the whole INR range. However, elimination of samples with INR >3 reduced the RMSE within INR <3 range to a value of 58 ng/ml in the derivation cohort and 49 ng/ml in the validation cohort. We suggest that our formula would be more accurate for evaluation of anti-FXa in cases where INR values are lower than three.

**Figure 4:**
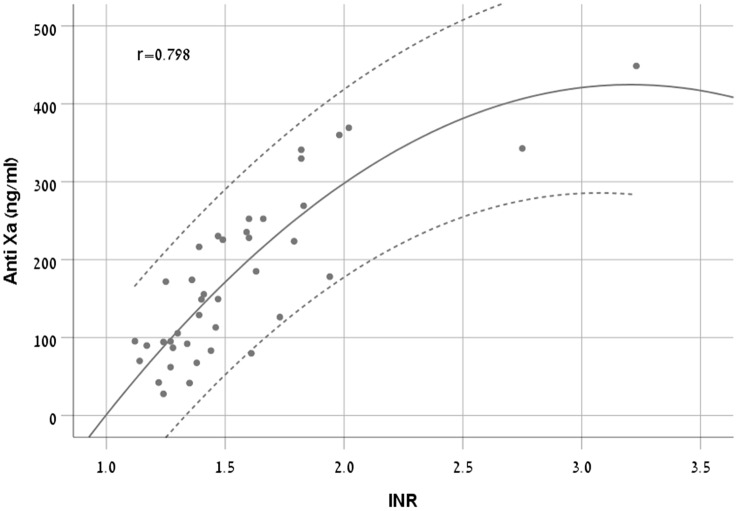
A scatter plot demonstrating the new equation with 95% CI in patients treated with apixaban.

There was a moderate to strong correlation between anti-FXa concentrations and INR (r = 0.798, p < 0.001). We chose Quadratic model as it had the highest R^2^ (0.711) (Equation 2, **Figure 4**).

$$\text{Anti-FXa}(\text{ng}/\text{ml})=-470.109+558.148\times \text{INR}-87.053\times {\text{INR}}^{2}$$

**Figure 5:**
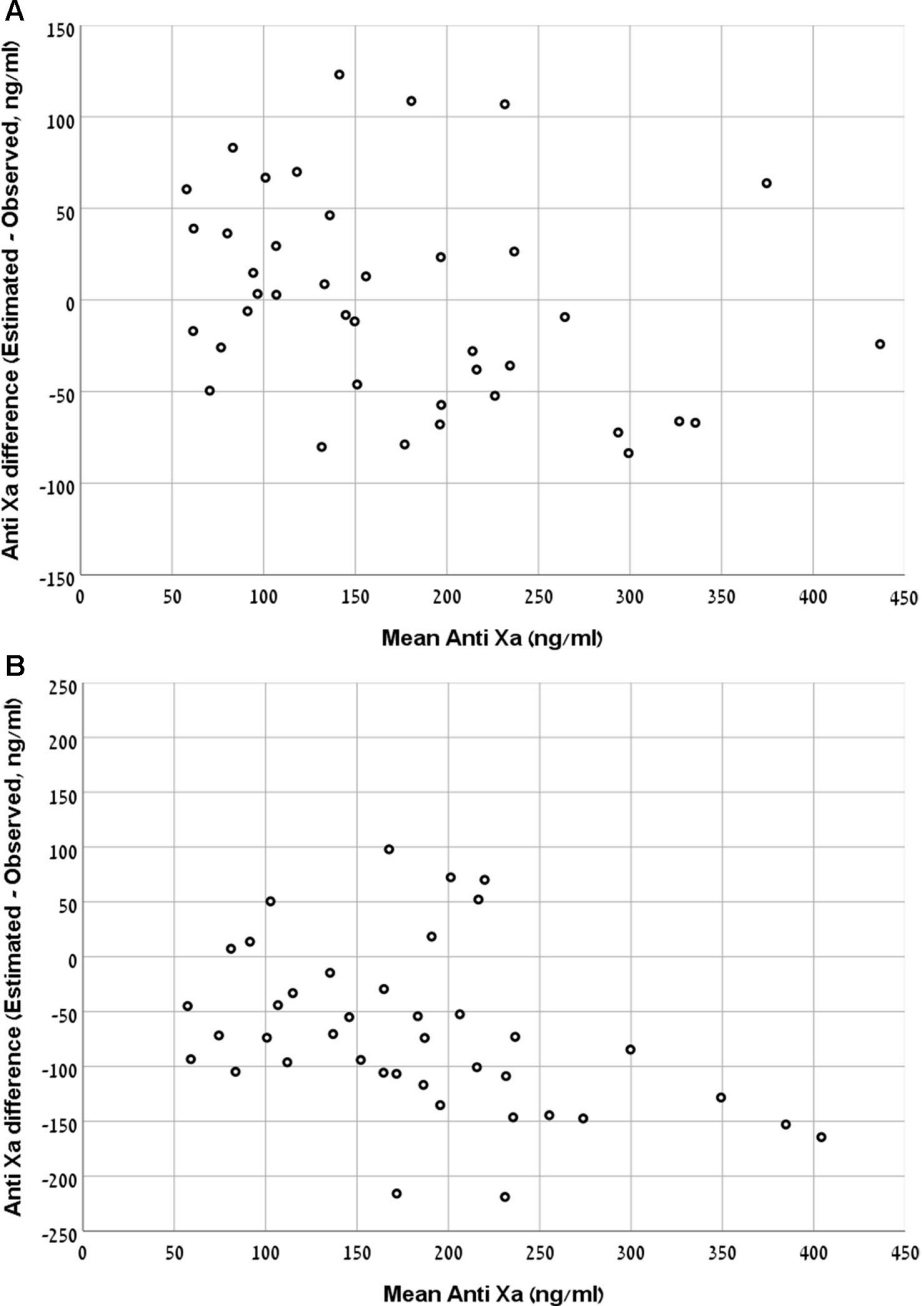
**(A)** Bland and Altman plot demonstrating the mean value of observed and expected Anti-FXa versus the difference between them in the apixaban derivation cohort. **(B)** Bland and Altman plot demonstrating the mean value of observed and expected Anti-FXa versus the difference between them in the apixaban validation cohort.

The RMSE of the model was 56 ng/ml in the derivation cohort and 102 ng/ml in the validation cohort. The Bland and Altman plot of the derivation and validation cohorts are presented in **Figures 5A, B**, respectively.

## Discussion

DOACs do not require routine monitoring as is necessary with Vitamin K antagonists (VKAs). However, laboratory measurement of blood levels or anticoagulant activity may be helpful in diverse clinical settings. There are qualitative type scenarios such as critical bleeding, urgent surgical reversal, and thrombolytic eligibility in acute stroke, where merely the presence or absence of drug effect is needed. On the other hand, there are other scenarios such as assessing for treatment failures and the clinical significance of drug-drug interactions that may require quantitative assessment i.e. estimates of the anticoagulant drug levels. In order to build our model, we used values of peak and trough out of the assumption that this conversion formula will be valid and utilized throughout the whole treatment period and will play a future role both in urgent and less urgent scenarios in centers without the ability to perform DOAC-specific anti-FXa assays.

Our results show that we were successfully able to introduce a novel formula, expressing the correlation between INR and anti-FXa for rivaroxaban treated patients.

$$\text{Anti-FXa}(\text{ng}/\text{ml})=e^{7.419-5.241x\frac{1}{\mathrm{INR}}}$$

This formula is valid throughout rivaroxaban whole INR sample range, but is significantly more precise for INR values below 3. This is clearly manifested by the RMSE values for rivaroxaban.

The calculated RMSE for the rivaroxaban group in the validation cohort (49 ng/ml) is even lower than this value for the derivation cohort (58 ng/ml), indicating a stable and validated model.

As described above, participants in each drug treatment group were further divided randomly into two equal groups. One group was used to build the model (derivation cohort), and the second group was used to validate it (validation cohort). As for apixaban, we could not device a validated model at the same level of certainty. The RMSE was almost doubled in the validation cohort (102 ng/ml) as compared to the derivation (56 ng/ml), indicating intolerable error and ruling out the use of the suggested formula (Equation 2) in clinical practice.

Pharmacokinetic studies showed that anti-FXa plasma levels vary widely from peak to trough. These studies defined expected steady state plasma anti-FXa levels at a given dose and a therapeutic indication, as the interval delineated by the 5^th^ percentile trough and the 95^th^ percentile peak plasma levels (**Table 2**). It must be emphasized that therapeutic ranges over which clinical outcomes are optimized have not been defined for these agents. By definition, the large majority of patients in steady state will have levels in this range at any time during treatment. For apixaban, which is administered twice daily, there is approximately a 2-fold difference between the median peak and trough concentrations. For rivaroxaban which is administered once daily, the difference is 8 to 10 fold (Frost et al., 2013; Samama et al., 2013; Kowalsk et al., 2014; Mueck et al., 2014; Bristol-Myers Squibb, 2018).

**Table 2:** Steady state peak and trough levels of apixaban and rivaroxaban (Samama et al., 2013; Bristol-Myers Squibb, 2018).

Drug	Dose	Indication	Median peak 5^th^ -95^th^ percentile	Median through 5^th^ -95^th^ percentile
Rivaroxaban	20mg/d	Prevention of stroke and systemic embolism in in NVAF	249 ng/ml (184−343)	44 ng/ml (12−137)
	15mg/d	Prevention of stroke and systemic embolism in in NVAF	229 ng/ml (178−313)	57 ng/ml (18−136)
	20mg/d	Treatment of DVT/PE and prevention of recurrent DVT/PE	270 ng/ml (189−419)	26 ng/ml (6−87)
Apixaban	5mgX2/d	Prevention of stroke and systemic embolism in in NVAF	171 ng/ml (91−231)	103 ng/ml (41−230)
	2.5mgX2/d	Prevention of stroke and systemic embolism in in NVAF	123 ng/ml (69−221)	79 ng/ml (34−162)
	5mgX2/d	Treatment of DVT/PE and prevention of recurrent DVT/PE	132 ng/ml (59−302)	63 ng/ml (22−177)
	2.5mgX2/d	Treatment of DVT/PE and prevention of recurrent DVT/PE	67 ng/ml (30−153)	32 ng/ml (11−90)

This information described above can give us a good perspective to understand the meaning of the RMSE values deviations. The rivaroxaban anti-FXa treatment range is wide and therefore, a deviation of approximately 50ng/ml may not be as significant as the same value for apixaban in light of its much narrower anti-FXa treatment range. From this point of view, considering the possible error, we would not recommend using apixaban’s formula to estimate anti-FXa by using INR values.

A study (Testa et al., 2016) evaluated the relationship of PT and aPTT versus three direct oral anticoagulants (apixaban, rivaroxaban, and dabigatran) concentrations measured by diluted thrombin time test calibrated for dabigatran and anti-FXa assays calibrated for rivaroxaban and apixaban. Four clinics were engaged in this study using a variety of PT reagents, anti- FXa assays, calibrators, and coagulometers. For rivaroxaban, the correlation (r) between the anti-FXa assay and the PT ranged from 0.91-0.73. Since results vary according to the reagent used, as was shown by *in vitro* and ex vivo studies (Barrett et al., 2010; Samama et al., 2013; Patel et al., 2015), this range apparently represents the use of different kits and instruments. The correlation resulted in our study (r = 0.899) corresponds with the higher value of the range presented by Testa, et al. This may be explained by a gained experience from understanding other studies that using a single type of reagent, calibrator, and coagulometer for both tests may provide better accuracy.

Testa et al. (2016) compared the anti-FXA and PT tests, and reported a good correlation between them, but apparently found it difficult to introduce an equation as they used simple linear regression. Since the data was not normally distributed and in order to meet the assumptions of the linear regression, we used natural logarithm transformation for anti-FXa values and reciprocal transformation for INR values. This method enabled us to convert our strong correlation value to a simple prediction model represented by a simple equation.

Another study (Douxfils et al., 2013) measured PT by two different reagents and obtained r value of 0.77. This value still falls within the range specified by Testa, et al. study and strengthen out the assumption that different reagents and analyzing methods may result in diverse correlation values. Furthermore, similar to Testa et al. study, Douxfils, et al. also used a linear regression. We are not convinced regarding its adequacy since the figures presented in that study show long right tail distribution, raising the need for using proper transformations to introduce a prediction model.

Scientific societies propose considering reversal agents or postponement of surgery at an anti-FXa level of 50 ng/ml in major bleeding or urgent surgery and at 30 ng/ml in cases of life-threatening bleeding or a high bleeding risk surgery (Levy et al., 2016; Godier et al., 2017). Our INR model shows a high correlation for rivaroxaban concentrations in the 30-50 ng/ml range. However, the number of samples is small and it would be improper to derive conclusions regarding the sensitivity of the formula for this specific population.

In summary, in our study, we sought to develop a model for using INR as a feasible, fast, and inexpensive screening test for estimating rivaroxaban and apixaban anticoagulation activity. Dincq et al., 2018 describes a rapid centrifugation protocol to reduce the turn-around time (TAT) of the laboratory measurement of rivaroxaban and apixaban plasma concentrations below 60 min. He also specifies other options such as reducing the period of reagent and control stabilization that may reduce the TAT to approximately 30 min. Implementation of these methods involves specific laboratory procedures, and thorough and continuous staff training. INR test, on the other hand, does not require special training. It is fast and results can be obtained within 5 min, which is significant in time critical situations. We demonstrated that utilizing a specific reagent and a specific work protocol, and performing suitable transformations, generates an equation which was validated and may be used for purposes of screening.

## Strengths and Limitations

The strength of our study, though involving only relatively small number of samples, is its accuracy. All participants were scrutinized carefully for their suitability to the study according to the strict inclusive criteria, reassuring a minimum of 4 days dosing, while supervising that the samples are withdrawn at their precise time. Since we used INR while previous studies tested the correlation of anti-FXa versus PT, the standardized INR presumably provides a greater cross lab validity and in view of our results, it has in fact statistically proven to be highly correlated with drug calibrated anti-Fxa assay. Some limitations of our work should be recognized. First, the International Council for Standardization in Haematology (ICSH) (Gosselin et al., 2018) recommends liquid chromatography-mass spectrometry/mass spectrometry (LC-MS/MS) as the gold standard test for measuring DOAC concentration. Due to its high degree of sensitivity, specificity, selectivity, and reproducibility, LC-MS/MS is the preferred method to evaluate DOAC pharmacokinetics in clinical development.

We did not use this technique at the time of our study, since it was not available in our lab. In fact, its widespread use in our country in the clinical setting is limited due to technique complexity and instrument availability. For this reason, we elected to use chromogenic anti- FXa assay as a reference. However, the ICSH guidelines point out that the drug-calibrated anti – Fxa demonstrated to be comparable to LC-MS/MS and thus, recommended as a suitable method to provide rapid quantitation of Anti -FXa agents. Second, the ICSH guidelines stress that the PT should not be expressed as INR in patients treated with DOACs, since the INR is based on VKA sensitivity. We completely agree with this undebatable scientific veracity statement. Nevertheless, our choice to use INR was driven by two reasons. INR is a cheap assay, already standardized, worldwide available even in labs in developing countries and most likely in rural areas of developed countries. INR is an adjustment for changes in PT reagents that allows for results from different labs to be compared. We assumed that using a standardized measurement in our research will provide greater cross lab uniformity. Third, we present in our study a conversion formula only for one FXa inhibitor, rivaroxaban. However, there are two other agents, apixaban and edoxaban, used in the clinical practice for which we could not provide a working formula. The formula built for apixaban could not been statistically validated and as for edoxaban, the drug has not been yet registered in Israel. Fourth, our results stem from a single type of kit to evaluate the PT and the Anti-FXa, while adhering to a specific laboratory work protocol and thus, we appreciate the assumption that different kits and a variety in lab procedures may provide different results. Therefore, it is imperative that future studies would test our model validity using a variety of kits manufactured by different companies, multiple instruments in multiple institutions. Lastly, we should also remark that in the rivaroxaban group for which a formula was derived and validated there were only AF patients and no VTE patients.

## Conclusions

Anti-FXa drug levels vary widely within individual patients from peak to trough and among different patients. Although routine laboratory monitoring of anticoagulant activity is not indicated, laboratory measurement may be desirable in special clinical settings and populations. In our study, we developed a formula to estimate the anticoagulant activity of rivaroxaban by obtaining INR values. We could not, however, retain the same success with prediction of apixaban coagulation activity, a fact that pretty well coincides and supported by the vast literature confirming that the PT test has insufficient sensitivity to be useful in patients taking apixaban or even for obtaining a crude estimate.

## Data Availability Statement

All datasets generated for this study are included in the manuscript/**Supplementary Files**.

## Ethics Statement

The studies involving human participants were reviewed and approved by Assaf-Harofeh Ethics Committee Trial No. 0168-17-ASF. The patients/participants provided their written informed consent to participate in this study.

## Author Contributions

FO, DB, NP, TZ-B, and MB (9th author) conceptualized and designed the study, drafted the initial manuscript, and approved the final manuscript as submitted. AM, TF-R, OG-J, and MB (8th author) carried out the initial analyses, reviewed and revised the manuscript, and approved the final manuscript as submitted.

## Conflict of Interest

The authors declare that the research was conducted in the absence of any commercial or financial relationships that could be construed as a potential conflict of interest.

## References

[B1] Barrett Y. C. Wang Z. Frost C. Shenker A. (2010). Clinical laboratory measurement of direct factor Xa inhibitors: anti-Xa assay is preferable to prothrombin time assay. Thromb. Haemost. 104, 1263–1271. 10.1160/TH10-05-0328 20978714

[B2] Bauer K. A. (2015). Targeted anti-anticoagulants. N. Engl. J. Med. 373, 569–571. 10.1056/NEJMe1506600 26095632

[B3] Bayer Pharma (2018). Rivaroxaban summary of product characteristics. Leverkusen, (Germany: Bayer Israel Ltd).

[B4] Bonar R. Favaloro E. J. Mohammed S. Ahuja M. Pasalic L. Sioufi J. (2016). The effect of the direct factor Xa inhibitors apixaban and rivaroxaban on haemostasis tests: a comprehensive assessment using *in vitro* and ex vivo samples. Pathology 48, 60–71. 10.1016/j.pathol.2015.11.025 27020211

[B5] Bristol-Myers Squibb S. R. L (2018). Eliquis^®^ summary of product characteristics. Italy: Pfizer Pharmaceuticals Israel Ltd.

[B6] Dale B. J. Ginsberg J. S. Johnston M. Hirsh J. Weitz J. I. Eikelboom J. W. (2014). Comparison of the effects of apixaban and rivaroxaban on prothrombin and activated partial thromboplastin times using various reagents. J. Thromb. Haemost. 12, 1810–1815. 10.1111/jth.12720 25196577

[B7] Dincq A. S. Lessire S. Pirard G. Siriez R. Guldenpfennig M. Baudar J. (2018). Reduction of the turn-around time for the measurement of rivaroxaban and apixaban: assessment of the performance of a rapid centrifugation method. Int. J. Lab. Hematol. 40 (6), e105–e108. 10.1111/ijlh.12870 29920946

[B8] Douxfils J. Chatelain C. Chatelain B. Dogné J. M. Mullier F. (2013). Impact of apixaban on routine and specific coagulation assays: a practical laboratory guide. Thromb. Haemost. 110, 283–294. 10.1160/TH12-12-0898 23765180

[B9] Douxfils J. Mullier F. Loosen C. Chatelain C. Chatelain B. Dogné J. M. (2012). Assessment of the impact of rivaroxaban on coagulation assays: laboratory recommendations for the monitoring of rivaroxaban and review of the literature. Thromb. Res. 130, 956–966. 10.1016/j.thromres.2012.09.004 23006523

[B10] Douxfils J. Tamigniau A. Chatelain B. Chatelain C. Wallemacq P. Mullier F. (2013). Comparison of calibrated chromogenic anti-Xa assay and PT tests with LC-MS/MS for the therapeutic monitoring of patients treated with rivaroxaban. Thromb. Haemost. 110, 723–731. 10.1160/TH13-04-0274 23846172

[B11] Eikelboom W. J. Connolly S. J. Bosch J. Dagenais G. R. Hart R. G. Shestakovska O. (2017). Rivaroxaban with or without aspirin in stable cardiovascular disease. N. Engl. J. Med. 377, 1319–1330. 10.1056/NEJMoa1709118 28844192

[B12] Francart S. J. Hawes E. M. Deal A. M. Adcock D. M. Gosselin R. Jeanneret C. (2014). Performance of coagulation tests in patients on therapeutic doses of rivaroxaban. A cross-sectional pharmacodynamic study based on peak and trough plasma levels. Thromb. Haemost. 111, 1133–1140. 10.1160/TH13-10-0871 24401946

[B13] Frost C. Nepal S. Wang J. Schuster A. Barrett Y. C. Mosqueda-Garcia R. (2013). Safety, pharmacokinetics and pharmacodynamics of multiple oral doses of apixaban, a factor Xa inhibitor, in healthy subjects. Br. J. Clin. Pharmacol. 76 (5), 776–786. 10.1111/bcp.12106 23451769 PMC3853536

[B14] Frost C. Song Y. Barrett Y. C. Wang J. Pursley J. Boyd R. A. (2014). A randomized direct comparison of the pharmacokinetics and pharmacodynamics of apixaban and rivaroxaban. Clin. Pharmacol. 13 (6), 179–187. 10.2147/CPAA.S61131 PMC4235474 25419161

[B15] Frost C. Wang J. Nepal S. Schuster A. Barrett Y. C. Mosqueda-Garcia R. (2013). Apixaban, an oral, direct factor Xa inhibitor: single dose safety, pharmacokinetics, pharmacodynamics and food effect in healthy subjects. Br. J. Clin. Pharmacol. 75 (2), 476–487. 10.1111/j.1365-2125.2012.04369.x 22759198 PMC3558798

[B16] Godier A. Dincq A. S. Martin A. C. Radu A. Leblanc I. Antona M. (2017). Predictors of pre-procedural concentrations of direct oral anticoagulants: a prospective multicentre study. Eur. Heart J. 38 (31), 2431–2439. 10.1093/eurheartj/ehx403 28821169

[B17] Gosselin R. C. Adcock D. M. Bates S. M. Douxfils J. Favaloro E. J. Gouin-Thibault I. (2018). International council for standardization in haematology (ICSH) recommendations for laboratory measurement of direct oral anticoagulants. Thromb. Haemost. 118, 437– 450. 10.1055/s-0038-1627480 29433148

[B18] Harenberg J. Krämer R. Giese C. Marx S. Weiss C. Wehling M. (2011). Determination of rivaroxaban by different factor Xa specific chromogenic substrate assays: reduction of inter assay variability. J. Thromb. Thrombol. 32, 267–271. 10.1007/s11239-011-0622-5 PMC3170460 21811937

[B19] Ikeda K. Tachibana H. (2016). Clinical implication of monitoring rivaroxaban and apixaban by using anti-factor Xa assay in patients with non-valvular atrial fibrillation. J. Arrhythmia 32, 42–50. 10.1016/j.joa.2015.08.001 PMC4759124 26949430

[B20] Kowalsk K. Nielsen J. Roy A. Thanneer N. Byon W. Boyd R. (2014). Apixaban exposure and anti-Xa activity in nonvalvular atrial fibrillation patients: an application of population PK/PD analysis. J. Pharmacokinet. Pharmacodyn. 41 (Suppl. 1), S19. 10.1007/s10928-014-9379-8

[B21] Kreutz R. Persson P. B. Kubitza D. Thelen K. Heitmeier S. Schwers S. (2017). Dissociation between the pharmacokinetics and pharmacodynamics of once-daily rivaroxaban and twice-daily apixaban: a randomized crossover study. J. Thromb. Haemost. 15, 2017–2028. 10.1111/jth.13801 28805299

[B22] Levy J. H. Ageno W. Chan N. C. Crowther M. Verhamme P. Weitz J. (2016). Subcommittee on control of anticoagulation. When and how to use antidotes for the reversal of direct oral anticoagulants: guidance from the SSC of the ISTH. J. Thromb. Haemost. 14 (3), 623–627. 10.1111/jth.13227 26911798

[B23] Mueck W. Stampfuss J. Kubitza D. Becka M. (2014). Clinical pharmacokinetic and pharmacodynamics profile of rivaroxaban. Clin. Pharmacokinet. 53, 1–16. 10.1007/s40262-013-0100-7 23999929 PMC3889701

[B24] Ofek F. Bar Chaim S. Kronenfeld N. Ziv-Baran T. Berkovitch M. (2017). International normalized ratio is significantly elevated with rivaroxaban and apixaban drug therapies: a retrospective study. Clin. Ther. 39 (5), 1003–1010. 10.1016/j.clinthera.2017.04.007 28476405

[B25] Patel J. P. Chitongo P. B. Czuprynska J. Roberts L. N. Patel R. K. Arya R. (2015). Normal prothrombin times in the presence of therapeutic levels of apixaban – *in-vivo* experience from king’s college hospital. Br. J. Haematol. 169, 138–153. 10.1111/bjh.13187 25312941

[B26] Samama M. M. Amiral J. Guinet C. Perzborn E. Depasse F. (2010a). An optimized, rapid chromogenic assay, specific for measuring direct FXa inhibitors (rivaroxaban) in plasma. Thromb. Haemost. 104, 1078–1079. 10.1160/TH10-03-0204 20806114

[B27] Samama M. M. Contact G. Spiro T. E. Perzborn E. Le Flem L. Guinet C. (2013). Laboratory assessment of rivaroxaban: a review. Throb. J. 11, 11–17. 10.1186/1477-9560-11-11 PMC3726412 23822763

[B28] Samama M. M. Martinoli J. L. Le Flem L. Guinet C. Plu-Bureau G. Depasse F. (2010b). Assessment of laboratory assays to measure rivaroxaban–an oral, direct FXa inhibitor. Thromb. Haemost. 103, 815–825. 10.1160/TH09-03-0176 20135059

[B29] Samuelson B. T. Cuker A. (2017). Measurement and reversal of the direct oral anticoagulants. Blood Rev. 31, 77–84. 10.1016/j.blre.2016.08.006 PMC5296289 27625113

[B30] Skeppholm M. Al-Aieshy F. Berndtsson M. Al-Khalili F. Rönquist-Nii Y. Söderblom L. (2015). Clinical evaluation of laboratory methods to monitor apixaban treatment in patients with atrial fibrillation. Thromb. Res. 136, 148–153. 10.1016/j.thromres.2015.04.030 25981142

[B31] Testa S. Legnani C. Tripodi A. Paoletti O. Pengo V. Abbate R. (2016). Poor comparability of coagulation screening test with specific measurement in patients receiving direct oral anticoagulants: results from a multicenter/multiplatform study. J. Thromb. Haemost. 14, 2194–2201. 10.1111/jth.13486 27566988

[B32] Tripodi A. Chantarangkul V. Legnani C. Testa S. Tosetto A. (2018). Interlaboratory variability in the measurement of direct oral anticoagulants: results from the external quality assessment scheme. J. Thromb. Haemost. 16, 565–570. 10.1111/jth.13949 29322630

[B33] Tripodi A. Padovan L. Veena C. Scalambrino E. Testa S. Peyvandi F. (2015). How the direct oral anticoagulant apixaban affects thrombin generation parameters. Thromb. Res. 135, 1186–1190. 10.1016/j.thromres.2015.03.032 25895845

[B34] Van Blerk M. Bailleul E. Chatelain B. Demulder A. Devreese K. Douxfils J. (2015). Influence of dabigatran and rivaroxaban on routine coagulation assays. A nationwide Belgian survey. Thromb. Haemost. 113, 154–164. 10.1160/TH14-02-0161 25231101

[B35] Van Blerk M. Bailleul E. Chatelain B. Demulder A. Devreese K. Douxfils J. (2017). Influence of apixaban on commonly used coagulation assays: results from the belgian national external quality assessment scheme. Int. Jnl. Lab. Hem. 39, 402–408. 10.1111/ijlh.12640 28304137

[B36] Van den Besselaar A. M. H. P. Poller L. Tripodi A. (1999). World health organization (WHO) guidelines for thromboplastins and plasma used to control oral anticoagulant therapy. WHO Tech. Rep. Ser. 889, 64–93.

[B37] Van Pelt L. J. Lukens M. V. Testa S. Chatelain B. Douxfils J. Mullier F. (2018). The DaXa-inhibition assay: a concept for a readily available, universal aXa assay that measures the direct inhibitory effect of all anti-Xa drugs. Thromb. Res. 168, 63–66. 10.1016/j.thromres.2018.04.024 29909093

