# Supplementary material for: International Normalized Ratio as a Screening Test for Assessment of Anticoagulant Activity for Patients Treated With Rivaroxaban or Apixaban: A Pilot Study
Source: Front Pharmacol. 2019 Oct 8;10:1177. doi: 10.3389/fphar.2019.01177 (PMC6792346; doi:10.3389/fphar.2019.01177)
Supplement: Supplementary file 1 [file Table_1.docx]

# Table 1A. Rivaroxaban and Apixaban treatment groups. Medication use and comorbidities on admission and comparison between learning and testing groups.

|  | Rivaroxaban group | | | | Apixaban group | | | |
| --- | --- | --- | --- | --- | --- | --- | --- | --- |
| Medication use - no. (%) | **Study Population (n= 40)** | **Learning**  **(n=20)** | **Testing**  **(n=20)** | **P Value** | **Study Population (n= 40)** | **Learning (n=20)** | **Testing**  **(n=20)** | **P Value** |
| Statins | 22 (55%) | 11 (55%) | 11 (55%) | >0.999 | 28 (70%) | 13 (65%) | 15 (75%) | 0.49 |
| Eltroxin | 6 (15%) | 3 (15%) | 3 (15%) | > 0.999 | 9 (22.5%) | 4 (20%) | 5 (25%) | > 0.999 |
| Laxatives | 5 (12.5%) | 2 (10%) | 3 (15%) | > 0.999 | 4 (10%) | 1 (5%) | 3 (15%) | 0.605 |
| Cardiovascular drugs | 37 (92.5%) | 19 (95%) | 18 (90%) | > 0.999 | 37 (92.5%) | 18 (90%) | 19 (95%) | > 0.999 |
| Antiplatelets | 5 (12.5) | 3 (15%) | 2 (10%) | >0.999 | 11 (27.5%) | 5 (25%) | 6 (30%) | 0.723 |
| Alpha Blockers (BPH) | 2 (10%) | 4 (20%) | 6 (15%) | 0.66 | 6 (15%) | 3 (15%) | 3 (15%) | > 0.999 |
| Glucocorticoids | 12 (30%) | 7 (35%) | 5 (25%) | 0.49 | 10 (25%) | 5 (25%) | 5 (25%) | > 0.999 |
| Allopurinol | 3 (7.5%) | 1 (5%) | 2 (10%) | > 0.999 | 4 (10%) | 1 (5%) | 3 (15%) | 0.605 |
| Inhaled Bronchodilators | 14 (35%) | 7 (35%) | 7 (35%) | > 0.999 | 11 (27.5%) | 5 (25%) | 6 (30%) | 0.723 |
| Antiarrhythmics | 18 (90%) | 16 (80%) | 34 (85%) | 0.66 | 36 (90%) | 19 (95%) | 17 (85%) | 0.605 |
| Nutritional Suppl. | 9 (22.5 %) | 7 (35%) | 2 (10%) | 0.127 | 9 (22.5%) | 4 (20%) | 5 (25%) | > 0.999 |
| Neuropathic pain drugs | 2 (5%) | 1 (5%) | 1 (5%) | > 0.999 | 7 (17.5%) | 3 (15%) | 4 (20%) | > 0.999 |
| Psychotropic drugs | 17 (42.5%) | 7 (35%) | 10 (50%) | 0.337 | 12 (30%) | 7 (35%) | 5 (25%) | 0.49 |
| Antacids | 25 (62.5%) | 10 (50%) | 15 (75%) | 0.1 | 22 (55%) | 10 (50%) | 12 (60%) | 0.525 |
| Antibiotics | 17 (42%) | 7 (35%) | 10 (50%) | 0.337 | 18 (45%) | 10 (50%) | 8 (40%) | 0.525 |
| Non - insulin antidiabetic drugs | 6 (30%) | 6 (30%) | 12 (30%) | > 0.999 | 18 (45%) | 7 (35%) | 11 (55%) | 0.204 |
| Insulin | 5 (10%) | 2 (15%) | 3 (12.5%) | > 0.999 | 11 (27.5%) | 5 (25%) | 6 (30%) | 0.723 |
| Comorbidity - no. (%) | | | | | | | | |
| IHD / CHF | 38 (95%) | 19 (95%) | 19 (95%) | >0.999 | 39 (97.5%) | 20 (100%) | 19 (95%) | > 0.999 |
| Previous CVA/TIA | 8 (20%) | 3 (15%) | 5 (25%) | 0.695 | 13 (32.5%) | 5 (25%) | 8 (40%) | 0.311 |
| Renal failure | 8 (20%) | 3 (15%) | 5 (25%) | 0.695 | 14 (35%) | 7 (35%) | 7 (35%) | > 0.999 |
| Infections | 8 (20%) | 3 (15%) | 5 (25%) | 0.695 | 9 (22%) | 4 (20%) | 56 (25%) | > 0.999 |
| Diabetes | 21 (52.5%) | 12 (60%) | 9 (45%) | 0.527 | 22 (55%) | 12 (60%) | 10 (50%) | 0.525 |
| Hyperlipidemia | 21 (52.5%) | 11 (52%) | 10 (50%) | > 0.999 | 17 (42.5%) | 8 (40%) | 9 (45%) | 0.749 |
| Hypothyroidism | 5 (12.5%) | 4 (20%) | 1 (5%) | 0.342 | 10 (25%) | 5 (25%) | 5 (25%) | > 0.999 |
| COPD/Asthma | 20 (50%) | 9 (45%) | 11 (55%) | 0.752 | 13 (32.5%) | 5 (25%) | 8 (40%) | 0.311 |
| Anemia | 8 (20%) | 4 (20%) | 4 (20%) | > 0.999 | 12 (30%) | 5 (25%) | 7 (30%) | 0.49 |

# Abbreviations:

# BPH (Benign Prostatic Hypertrophy), IHD (Ischemic Heart Disease), CHF (Congestive Heart Failure),

# CVA (Cerebro-Vascular Accident), TIA (Transient Ischemic Attack), COPD (Chronic Obstructive Pulmonary Disease).
